# Variation ontology: annotator guide

**DOI:** 10.1186/2041-1480-5-9

**Published:** 2014-02-17

**Authors:** Mauno Vihinen

**Affiliations:** 1 Department of Experimental Medical Science, Lund University, BMC D10, SE-22184 Lund, Sweden

**Keywords:** Variation ontology, Annotation instructions, Systematics, Variation effects, Mutation, Ontology

## Abstract

**Background:**

Systematic representation of information related to genetic and non-genetic variations is required to allow large scale studies, data mining and data integration, and to make it possible to reveal novel relationships between genotype and phenotype. Although lots of variation data is available it is often difficult to use due to lack of systematics.

**Results:**

A novel ontology, Variation Ontology (VariO http://variationontology.org), was developed for annotation of effects, consequences and mechanisms of variations. In this article instructions are provided on how VariO annotations are made. The major levels for description are the three molecules, namely DNA, RNA and protein. They are further divided to four major sublevels: variation type, function, structure, and property, and further up to eight sublevels. VariO annotation summarizes existing knowledge about a variation and its effects and formalizes it so that computational analyses are efficient. The annotations should be made on as many levels as possible. VariO annotations are made in reference to normal states, which vary for each data item including e.g. reference sequences, wild type properties, and activities.

**Conclusions:**

Detailed instructions together with examples are provided to indicate how VariO can be used for annotation of variations and their effects. A dedicated tool has been developed for annotation and will be further developed to cover also evidence for the annotations. VariO is suitable for annotation of data in many types of databases. As several different kinds of databases are in a process of adapting VariO annotations it is important to have guidelines to guarantee consistent annotation.

## Background

Variations have different effects and mechanisms. To capture and describe the character of the variations at DNA, RNA and protein level, the Variation Ontology (VariO) was developed [1]. VariO annotations allow systematic descriptions which can be used e.g. for searches of complex queries also simultaneously from several databases. Systematic descriptions have several benefits especially for computational searches and analyses and for software development.

Variation information has been collected to various databases. Locus specific databases (LSDBs) are for individual genes or diseases and usually curated by experts in the genes and diseases. There are currently thousands of LSDBs available, mainly on the LOVD database management system [2]. Central databases like SwissProt [3] and ClinVar [4] contain information on large numbers of genes and/or proteins and variations in them. Additional types of variation databases include national or ethnic databases such as ETHNOS [5], chromosomal variation databases such as The Database of Genomic Variants archive (DGVa) [6], variation frequency databases including FINDbase [7], and databases dedicated on certain types of variations or for an effect or mechanism, such as ProTherm, a database for protein stability affecting variations [8]. Benchmark databases, such as VariBench [9], are dedicated for providing gold standard datasets for method developers and assessors. All these databases would benefit from systematic descriptions. Human Genome Variation Society (HGVS) nomenclature is widely used for naming variations [10], however additional systematics would be needed. HGVS and Human Variome Project (HVP) have released a number of recommendations [11], also for increased systematics, including recommendation to use VariO annotations [12]. The recent recommendations for LSDB establishment and curation emphasize the importance of systematics [13,14]. The goal of the GEN2PHEN project was to develop tools, data models and solutions for this domain [15].

The HGVS variation nomenclature is a systematic naming convention that is in use in some journals and numerous databases. It provides guidelines and naming for almost all variation types based on reference sequences. The Mutalyzer tool can generate the names automatically and performs a number of consistency checks [16].

VariO is intended for the description of what is changed in the variant in comparison to the normal or wild type. Thus, it does not describe the properties of the wild type. The annotations are made in comparison to a reference, which varies depending on the annotated property including e.g. reference sequences, reference states such as wild type enzyme activity and normal kinetic properties. The application area of VariO is in describing effects, consequences and mechanisms in diverse data sources. These include all the different types of variation databases mentioned above. In addition, it can benefit variation naming services [16], LSDB management systems [2], data integrators, journals etc. As VariO has been described previously [1], the goal of this annotator guide is to explain how annotations are made and used.

### VariO design principles

VariO terms have three biological molecules, DNA, RNA and protein, as the starting point. All these have four major sublevels: *variation type*, *function*, *structure* and *properties* with more detailed sublevels. There are altogether 8 levels of terms. The terms have a clear hierarchy and the organization of terms for DNA, RNA and protein has a similar and coherent layout whenever appropriate. The terms for the three molecular levels are consistent and related terms are used for related features at different levels. Suggestions for additional terms and updates can be sent to the ontology developers.

**Figure 1:**
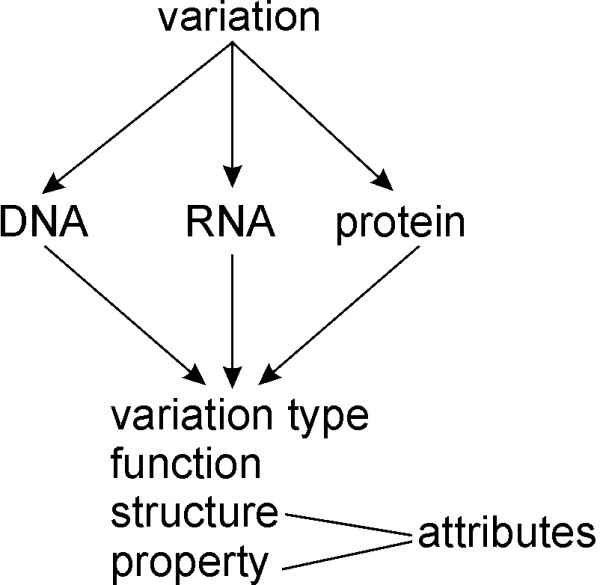
**General structure of VariO.** The ontology is designed for annotation of effects, consequences and mechanisms of variations at the three molecular levels, DNA, RNA and protein. Each of these has further terms on four major sublevels: variation type, function, structure and property. Attributes are used to modify terms at structure or property levels to further define the terms.

For visualization of terms, their definitions, relationships and paths, the AmiVariO browser is available at the VariO website. Figure 1 indicates how the levels are organized. When possible, variations are explained at DNA, RNA and protein levels. Each of these has sublevels, out of which structure and property levels can be further modified with attribute terms. The hierarchy of the terms has been designed to allow for a versatile and flexible annotation.

**Figure 2:**
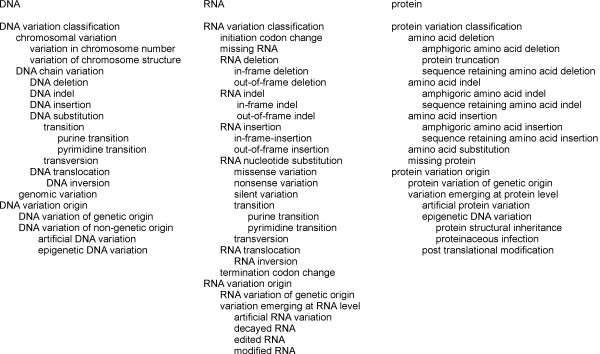
**Variation types for terms with genetic or non-genetic origin.** The variation type annotations are made based on whether the variation is of genetic or non-genetic origin. Genetic terms are used for alterations originating from the genetic material of the organism (DNA or RNA, depending on the organism), while terms with non-genetic origin are either artificial or originated from the processing of RNA or protein molecules without change in the corresponding DNA.

VariO is intended for the description of all kinds of variations and situations. In Figure 2 the distinction between terms of genetic and non-genetic origin is shown. Terms with genetic origin describe changes either in DNA or inherited from it to RNA and protein levels. The non-genetic terms, called variations emerging at the RNA or protein level, are for either biological or artificial modifications that originate at the RNA or protein level. For example, RNA editing modifies a synthetized RNA chain and the variations are not coded in DNA.

To keep the ontology compact, modifier attributes form the fourth major level. These terms are used to modify the terms at the other levels. For example, quantity terms are used to modify other terms when the effect is increased, decreased or is missing the quantity of the parameter, or when it is not changed. Instead of having separate terms for describing *increase* or *decrease* of a feature, existing terms can be modified with attribute terms. This way the number of terms could be reduced considerably.

VariO terms should be combined with other systematics and ontologies. Evidence Ontology (ECO) terms [17] are used to describe the methods with which the annotations were obtained.

VariO aims at describing any effect, consequence and mechanism, at any organism. The variations can be of genetic or non-genetic origin. The size of the variation does not matter, anything ranging from nucleotide or amino acid changes to chromosome or genome duplications can be annotated. However, the annotations are position based, even if the position means e.g. an entire chromosome.

### Example of VariO annotation

This example highlights a number of aspects of annotations with VariO. The annotations are used to explain additional features of the instances in a database in a systematic way. It may be tiring for a human reader to see the same concept every time it is mentioned, however, for computational analyses it is a blessing and facilitates fast searches. For the annotation we are developing a tool called VariOtator [18].

Here is a real life example of variation in the *AIRE* gene leading to the autoimmune polyendocrine syndrome type 1 (APS-1) also called for APECED disease (**a**utoimmune **p**oly**e**ndocytopathy-**c**andidiasis-**e**ctodermal **d**ystrophy), an autoimmune polyendocrine syndrome. AIRE, autoimmune regulator, is a transcriptional regulator of tissue-specific antigens. Variations affect the regulation, leading to the production of self-reactive antigens. The T > C variation leading to a L to P substitution in the homogeneous staining region (HSR) domain is disease causing [19] (AIREbase [20,21] entry A0087). The functional and other aspects of APECED-causing variations were further studied in [22].

The genomic variant g.4789 T > C in the IDbase reference sequence D0003 (http://structure. bmc.lu.se/cgi-bin/fetch_idrefseq.cgi?ac = D0003&format = embl, cross-reference to EMBL:AB006682) is annotated as follows.

VariO:0128 variation affecting DNA

VariO:0129 DNA variation type

VariO:0322 DNA variation classification

VariO:0135 DNA chain variation

VariO:0136 DNA substitution

VariO:0313 transition

VariO:0314 pyrimidine transition

The variation is of genetic origin

VariO:0128 variation affecting DNA

VariO:0129 DNA variation type

VariO:0127 DNA variation origin

VariO:0130 DNA variation of genetic origin

There is a pyrimidine transition of genetic origin. The effect to RNA sequence (IDbase reference sequence C:0003 http://structure.bmc.lu.se/cgi-bin/fetch_idrefseq.cgi?ac = C0003& cross referenced to EMBL; AB006682) is, similar at DNA level, a pyrimidine transition, which causes a missense variation.

VariO:0297 variation affecting RNA

VariO:0306 RNA variation type

VariO:0328 RNA variation classification

VariO:0312 RNA substitution

VariO:0313 transition

VariO:0314 pyrimidine transition

VariO:0308 missense variation

On the protein level the reference sequence is UniProt entry O43918. A variation has different annotations at different levels. In this example, the amino acid substitution at protein level is annotated as DNA substitution on DNA level, and on RNA level as RNA nucleotide substitution of type missense variation.

The annotations are richer on protein level as the effects of the variation affect protein function, structure and properties.

VariO:0002 variation affecting protein

VariO:0012 protein variation type

VariO:0325 protein variation classification

VariO:0021 amino acid substitution

The protein variation is due to change at the DNA level.

VariO:0002 variation affecting protein

VariO:0323 protein variation origin

VariO:0013 protein variation of genetic origin

This variant was shown to prevent transactivation, a protein information transfer function.

VariO:0002 variation affecting protein

VariO:0003 variation affecting protein function

VariO:0011 effect on protein information transfer

The annotations for the structure include predicted effects on the protein secondary and tertiary structure. Introduction of a proline in the middle of α-helix affects the protein fold leading to conformational change.

VariO:0002 variation affecting protein

VariO:0060 variation affecting protein structure

VariO:0064 effect on protein 3D structure

VariO:0070 effect on protein tertiary structure

VariO:0079 effect on protein secondary structural element

VariO:0080 effect on protein helix

VariO:0082 effect on right handed protein helix

VariO:0085 effect on alpha helix

VariO:0002 variation affecting protein

VariO:0060 variation affecting protein structure

VariO:0064 effect on protein 3D structure

VariO:0070 effect on protein tertiary structure

VariO:0073 effect on protein fold

VariO:0074 protein conformational change

Properties are used for annotating various characteristics. As many properties should be annotated as data is available. The variation is disease-causing, which is indicated by the pathogenicity association attribute. In the case of attributes, only the relevant attribute, not path to it is given. Attributes can be used to modify structure and property terms.

VariO:0002 variation affecting protein

VariO:0032 variation affecting protein property

VariO:0047 association of protein variation to pathogenicity; VariO:0294 disease causing

The variant affects transactivation inactivating the protein function. The property term (effect on protein activity) is again modified by the attribute (missing).

VariO:0002 variation affecting protein

VariO:0032 variation affecting protein property

VariO:0053 effect on protein activity; VariO:292 missing

Effects to protein interaction can be described in detail. Interaction attribute terms were derived from the Protein Interaction ontology [23] but modified for the purpose of VariO.

VariO:0002 variation affecting protein

VariO:0032 variation affecting protein property

VariO:0058 effect on protein interaction; VariO:0292 missing

The variant prevents AIRE homomultimerization, which is required for the transactivation activity, and is essential for the interaction with nuclear dots and cytoplasmic filaments. This can be further described by the attributes as follows for the homomultimerization, i.e. interaction with another protein molecule.

VariO:0232 variation attribute

VariO:0236 interaction

VariO:0262 interactor

VariO:0273 biopolymer

VariO:0277 proteinand the interaction with nuclear dots and cytoplasmic filaments are annotated as effects on protein complexes.

VariO:0232 variation attribute

VariO:0236 interaction

VariO:0262 interactor

VariO:0267 complex

VariO:0269 protein complex

Even more detailed descriptions would be possible with VariO, however, in this case as details are missing, the annotations remain somewhat shallow. For instance, interaction physical forces cannot therefore be annotated.

The variant alters the subcellular localization of the protein. Terms for the actual change to the compartment targeting are not provided as they have not been systematized.

VariO:0002 variation affecting protein

VariO:0032 variation affecting protein property

VariO:0033 effect on protein subcellular localization

This example highlights how the experimental and predicted results can be explained at multiple levels. Note that e.g. details of protein function are further explained by the property terms. Above, attributes are used as simple modifiers of quantity for protein activity and for disease causality, but these modifiers allow also more elaborate annotations as for interactions. The VariO annotation is modular and therefore any number of terms can be used, whatever is needed to capture the type and effects of a variant. More examples are available at http://variationontology.org/examples.shtml.

### How to get started

**Figure 3:**
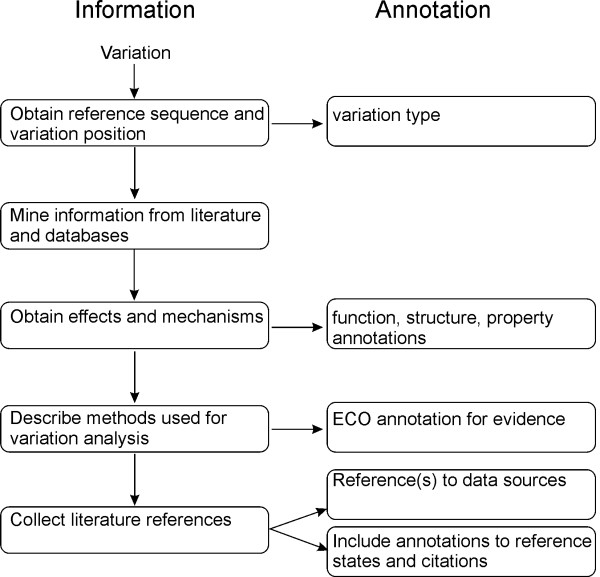
**Flowchart for annotation with VariO.** All existing information for the variation should be available when starting annotation. Reference sequence is needed to indicate the position of the variation. With this information can be generated the variation type annotation. The effects on function, structure and property are annotated based on the obtained information. The methods used for obtaining the results are indicated by Evidence Codes terms. Literature and database references are used to indicate the origin of the annotated data. It is essential that the database using VariO annotations provides information about all relevant reference states such as reference sequence and wild type protein properties.

When annotating a variant one should combine all the existing information about the variant and its effects. The steps from the variation identification to a functional annotation are depicted in Figure 3. In case of contradictory results try to obtain consensus. Databases should not reflect personal opinions, therefore the annotations have to be according to the approved standards in the field. Once the data is available all relevant aspects should be described. This may require annotations at several sublevels, for example variation types can be further defined at structural level.

Once the data is available the annotation process is relatively straightforward. The annotation tool, VariOtator, can be used for generating annotations. In the future, we will provide a full annotation tool covering also evidence. While reading the literature find which methods have been used to obtain the results as they are needed for Evidence Code annotation. For ECO annotations, the methods used for obtaining the results have to be taken from the literature. Literature and database references describing the variant should be provided in the database.

### VariO annotation

#### Terms at major sublevels

There are four major sublevels. Variation type is to explain the type of variation. These annotations can be generated from systematic HGVS names [9]. The annotation terms do not provide the actual sequence variation information, the change(s) at nucleotide or amino acid level, since HGVS names are for their systematic description. Instead they provide genetic terms for the type of the variation. This is often useful for human users as the HGVS names can be quite complex and one needs to be familiar with the naming conventions to be able to decipher them correctly.

Function terms indicate the biological function affected by the variation. The function is annotated on the level(s) where affected. So, effects on protein function are only described at protein level. However, if the effect is e.g. on RNA level then annotation is made on that level.

**Figure 4:**
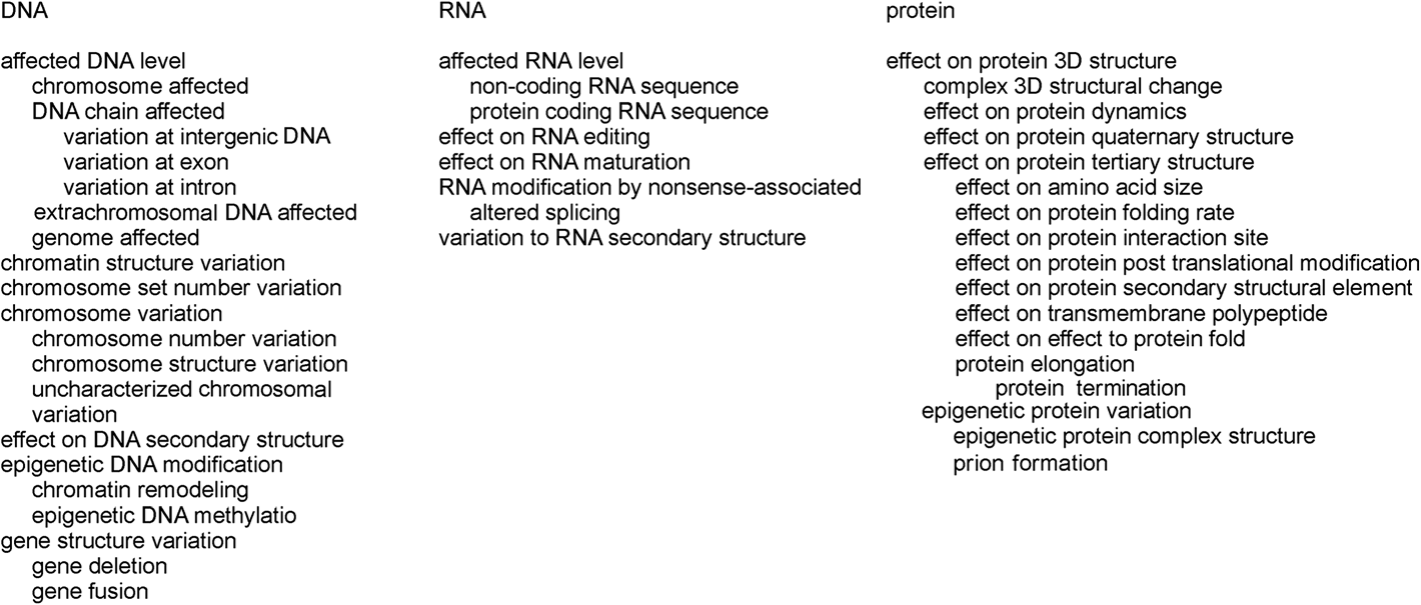
**Organization of major structure terms at different levels.** Some sublevels are indicated.

Structure annotations allow for an elaborate description of the effects to the DNA, RNA or protein structure. Due to the characteristics of the structures at the three levels the terms vary (Figure 4). The most detailed annotations can be made for protein changes. These terms can be modified with attributes, when necessary/if desired.

Property terms have great differences between the molecular levels. Some terms are common at two or three levels including sequence conservation, association of the variation to pathogenicity, and interaction. Effects to abundance, degradation and stability are shared by RNA and protein levels. In addition, there are protein specific terms.

#### Full path for terms

The VariO annotations should be presented as full paths from the branch to the root as there is thereby more information and disambiguation can be avoided. Annotations consist at least of the identifier of the term, e.g. VariO:0022 which is composed of two parts, VariO: in the beginning indicates what ontology is used and then the four digit number is for the individual term. Usually also the name of the term is added, in this example “amino acid indel”. All terms in VariO have definitions as well as relationships to other terms up and down in the hierarchy.

Exceptions to the rule of full path for terms are attributes for conservation, pathogenicity association, and quantity change. As there is just one single sublevel for these terms they are used without the path. Since there are several sublevels for interaction attributes they have to be provided in full.

#### Provide all necessary annotations

Annotations are most useful when they are rich and detailed. This is a true strength of VariO which facilitates very detailed descriptions and equally detailed searches for certain kinds of cases.

The more data there are the more annotations can be made. The VariO concepts are balanced between practical application and existence of details. It would be possible to have even more detailed terms, however, cases in which they could be used might be very limited. The level of detail has been adjusted so that all variants and effects can be explained to a certain extent. The ontology will be updated in the future and new terms can be added e.g. when new technologies are introduced and more details are provided.

#### Reference states

VariO describes the consequences and mechanisms in relation to a reference state. In the case of sequences, the recommended references are Locus Reference Genomic (LRG) [24] entries, when available. In the case of biological functions the reference is the property of the wild type molecule. These references should be available in the databases and explained in detail.

#### Use attributes as modifiers

Terms at the structure and property levels can be modified by attributes. Note that attributes are not used to specify *variation type* and *function* terms. Attributes have four major levels. Conservation attributes explain what kind of change has been introduced (conserved, covariant or nonconserved). Interaction terms describe effects to interactions. Pathogenicity association clarifies the relationship of the variation to pathogenicity, or not. Quantity change attributes define the direction and extent of the change and contain the terms VariO:0290 decreased, VariO:0291 increased, VariO:0292 missing, and VariO:0140 not changed. For the use of interaction annotations see the example above.

#### Evidence codes

The annotations should be accompanied by terms from another ontology, Evidence Codes. These ECO terms describe the (experimental) method used to obtain the annotated feature. This will allow users to evaluate the quality of data items. The term should be used at the level where the description is applicable. ECO terms are used without the full path of the terms.

To the example above some ECO terms can be added as follows. The variant protein is inactive. This was verified by two-hybrid enzyme assay with luciferase as reporter. To the annotation is added ECO:0000049 reporter gene assay evidence. The variant prevents interactions required for the functional protein complex formation. This was revealed with gel filtration fractionation. The term to be included is in this case ECO:0000156 protein separation evidence. The variant has also effect on protein localization, which is annotated as ECO:0000007 immunofluorescence evidence. These specification terms are linked to the relevant VariO annotations.

#### Annotation for variants of genetic and non-genetic origin

The variation may have originated on the level they are described or be inherited from changes in DNA. Variants that can be derived from changes on DNA are annotated with variation types of genetic origin. The terms for VariO:0146 DNA variation of non-genetic origin, VariO:0333 variation emerging at RNA level, and VariO:0024 variation emerging at protein level describe changes not originating from DNA change, instead e.g. epigenetic DNA and protein variations as well as edited RNA (Figure 2). These include also artificial variations on all three molecular levels, because VariO is intended to allow annotation of variations e.g. originating from protein engineering.

#### Combine different systematics for even richer annotations

VariO is a specific ontology with a well-defined application area. It could and should be used together with other systematic descriptions related to variations. These include, systematic gene names provided by the HUGO Gene Nomenclature Committee (HGNC) [25], the HGVS variation nomenclature [9], reference sequences, preferably LRGs [24]. For the description of clinical features the Human Phenotype Ontology (HPO) [26] and Elements of Morphology [27] can be used. Evidence Codes ontology annotations should be added to further clarify the terms by indicating the method used to obtain the results as described above.

#### Minimum annotation

As VariO is position specific the minimum annotation would include the details at DNA, RNA and protein level, when changes on all three levels appear. However, it is highly recommended to include additional information at other levels, although already systematic variation type descriptions are useful.

#### Include information sources

The annotations need to be supplemented with information about the reference states as well as references to the sources of information such as literature, database or prediction. Database and literature references should be linked to the original sources when annotating databases.

#### Version number

Databases using VariO annotations, and other ontologies, should state which version of the ontology has been utilized. As ontologies are under constant improvement it is important to indicate according to which set of terms the annotations have been made. This information applies to the entire database and thus needs to be provided just once.

### Annotation tool

We are developing dedicated software for providing annotations. VariOtator is available at [17] and currently generates variation type annotations based on variation descriptions based on HGVS names for variations as well as structure, function and property annotations. In the future, the tool will allow the Evidence Code annotations to be incorporated.

## Competing interests

The author declares that he has no competing interests.
